# Fibrin Glue for Sutureless PreserFlo Surgery: A Case Series

**DOI:** 10.7759/cureus.100185

**Published:** 2025-12-27

**Authors:** Cadric Gunaratnam, Ario Wilson-Pogmore, Susan Zhang, Stephen O'Hagan

**Affiliations:** 1 Department of Ophthalmology, Cairns Base Hospital, Cairns, AUS; 2 School of Medicine, University of Queensland, Brisbane, AUS; 3 School of Medicine, James Cook University, Cairns, AUS

**Keywords:** fibrin glue, glaucoma, preserflo, sutureless surgery, trabeculectomy

## Abstract

Purpose

To evaluate the efficacy of sutureless PreserFlo MicroShunt insertion using fibrin glue for conjunctival closure and scleral band fixation in patients with advanced glaucoma in Cairns, Australia.

Methods

A retrospective case series evaluated seven patients undergoing urgent PreserFlo MicroShunt insertion at Cairns Hospital (March 2023 to November 2024). VeraSeal fibrin glue was used for conjunctival closure, with scleral band fixation in three cases. Pre- and post-operative intraocular pressure (IOP), visual acuity (VA), and ocular hypotensive medications were measured. Primary outcomes included intraocular pressure (IOP) and glaucoma medication reduction at six months and non-routine post-operative reviews within the first month. Secondary outcomes were complications and revision surgery needs. Success was defined as >20% IOP reduction at six months.

Results

Seven patients (mean age 74 years, range 54-87 years; three neovascular glaucoma, three primary open-angle glaucoma, one aphakic glaucoma) were included. Mean IOP decreased from 34.9±16.5 mmHg to 11.3±3.8 mmHg at six months. Medications reduced from a mean of 3.3 (range 3-5) to 0.1 (range 0-1). Non-routine visits averaged 3.7 (range 3-4). Complications included transient hypotony (n=1), tube exposure (n=1, managed with fibrin glue), macrohyphema (n=3), and pre-existing Snuff syndrome (n=2). No glue-related complications occurred. Scleral band fixation cases showed well-formed blebs without extrusion.

Conclusions

Sutureless PreserFlo MicroShunt with fibrin glue and scleral band fixation effectively reduces IOP and medication use with minimal complications and follow-up. Further studies are needed to validate this approach.

## Introduction

The PreserFlo MicroShunt (Glaukos, Ontario, Canada) is a minimally invasive glaucoma drainage device designed to optimize intraocular pressure (IOP) control while reducing reliance on IOP-lowering medications [1]. Compared with trabeculectomy, PreserFlo offers similar IOP reduction but fewer postoperative complications and procedural interventions [1]. Fibrin glue has been reported to be a safe alternative to sutures in glaucoma surgery, decreasing operative time and conjunctival inflammation without compromising IOP outcomes [2,3]. To date, no published studies have evaluated the role of fibrin glue in PreserFlo implantations. This case series presents the first reported use of fibrin glue for conjunctival closure in PreserFlo insertion, assessing its efficacy and safety in seven patients, and describes the first documented use of scleral band fixation to achieve a completely sutureless approach for use in PreserFlo surgery in Cairns, Australia. The primary objectives of this preliminary case series were to establish and evaluate (1) the efficacy of completely sutureless PreserFlo MicroShunt implantation using fibrin glue for conjunctival closure in achieving ≥20% IOP reduction and reduction in medication burden at six months and (2) the early postoperative course and complications (i.e., number of non-routine visits in the first month). Secondary objectives were to report intraoperative time savings, complications, and the need for revision surgery.

## Materials and methods

Study design

This study was approved by the Human Research Ethics Committee of Far North Queensland (HREC/2025/QCH/119768) and conducted in accordance with the ethical standards of the Declaration of Helsinki. The study included seven patients who underwent PreserFlo MicroShunt insertion at Cairns Hospital from March 2023 to November 2024. All the surgeries were performed by a single surgeon (S.O.). We identified cases from surgical records and extracted data on demographics, visual acuity (VA), IOP, and ocular hypotensive medications from the electronic records.

All patients had advanced or end-stage glaucoma requiring urgent surgery because of failed maximal medical treatment. Indications included inadequate IOP control despite maximal medical therapy (n=3), progressive glaucoma with severe vision impairment or visual field loss (n=2), and ocular pain from chronic uncontrolled IOP unresponsive to systemic analgesia (n=2). All patients had primary or secondary glaucoma with advanced disease and were classified as having operative emergencies.

Surgical technique

**Figure 1:**
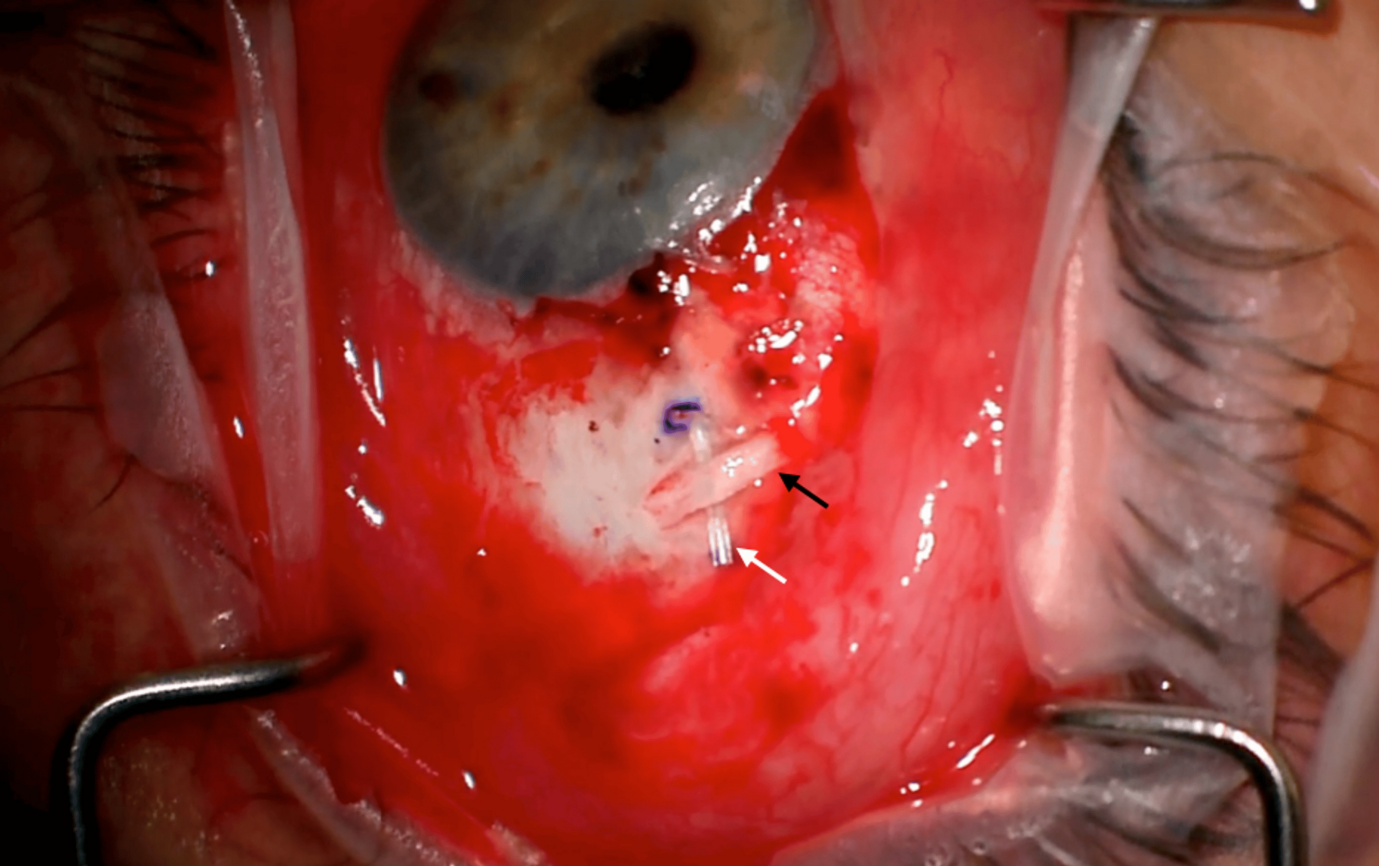
Scleral Band Fixation Technique Intraoperative photograph illustrating a scleral band 3 mm × 1 mm (black arrow) fashioned 4 mm posterior to the limbus over the distal end of the PreserFlo tube (white arrow).

A sutureless technique was used in all patients. A superotemporal limbal conjunctival peritomy was performed, followed by blunt dissection beneath the Tenon’s capsule to the bare sclera. Mitomycin-C (0.02%)-soaked pledgets were applied to the scleral bed for 2 min, removed, and the area was irrigated with balanced salt solution (BSS). A sclerostomy was performed 3 mm posterior to the limbus using a modified knife. A PreserFlo MicroShunt was inserted into the anterior chamber via sclerostomy, with the wings positioned inside the tunnel entrance. In three cases, a scleral band (3 mm × 1 mm) was fashioned 4 mm posterior to the limbus (Figure 1) to secure the distal end of the shunt to the sclera without sutures. VeraSeal fibrin glue (Instituto Grifols S.A., Barcelona, Spain) was initially applied with thrombin to the limbal sclera and conjunctiva, followed by fibrinogen to the underside of the limbal section of the fornix-based conjunctival flap. The conjunctival flap was held in place for 2 min to bond. The anterior chamber was reformed with BSS via paracentesis, and the conjunctival bleb was inspected for leaks using 2% fluorescein. An intracameral injection (cefazolin, 1 mg/0.1 ml) and an inferior fornix subconjunctival injection (dexamethasone, 4 mg/ml) were administered at the conclusion of the procedure.

Follow-up and outcomes

Patients were routinely reviewed post-operatively at one day, one week, one month, and six months. The primary outcomes included IOP reduction at six months, number of non-standard reviews in the first month, and reduction in glaucoma medications. The secondary outcomes were complications and requirements for surgical revision. Success in lowering IOP was defined as >20% reduction in pre-operative IOP at six months.

## Results

Seven patients (four men, three women) with a mean age of 74 years (range, 54-87 years) were reviewed (Table 1). Diagnoses included neovascular glaucoma (NVG; n=3), primary open-angle glaucoma (POAG; n=3), and aphakic glaucoma (n=1). Indications for surgery included inadequate IOP control despite maximally tolerated medical therapy (n=3), progressive glaucoma with severe vision impairment or visual field loss (n=2), and/or ocular pain from chronic uncontrolled IOP not responding to systemic analgesia (n=2). None had prior glaucoma filtration surgeries; five had previous cataract surgery (four pseudophakic, one aphakic). The mean preoperative IOP was 34.9±16.5 mmHg, with patients using a mean of 3.3 glaucoma medications.

**Table 1:** Summary of Patient Demographics (n=7) Data are expressed as number (%) or mean (range). POAG: primary open-angle glaucoma, NVG: neovascular glaucoma.

Characteristics	Values
Sex	-
Male	4 (57)
Female	3 (43)
Mean Age (range)	74 years (54–87 years)
Ethnicity	-
Indigenous	1 (14)
Caucasian	6 (86)
Glaucoma type	-
POAG	3 (42)
NVG	3 (42)
Aphakic	1 (14)
Lens Status	-
Phakic	2 (29)
Pseudophakic	4 (57)
Aphakic	1 (14)

**Figure 2:**
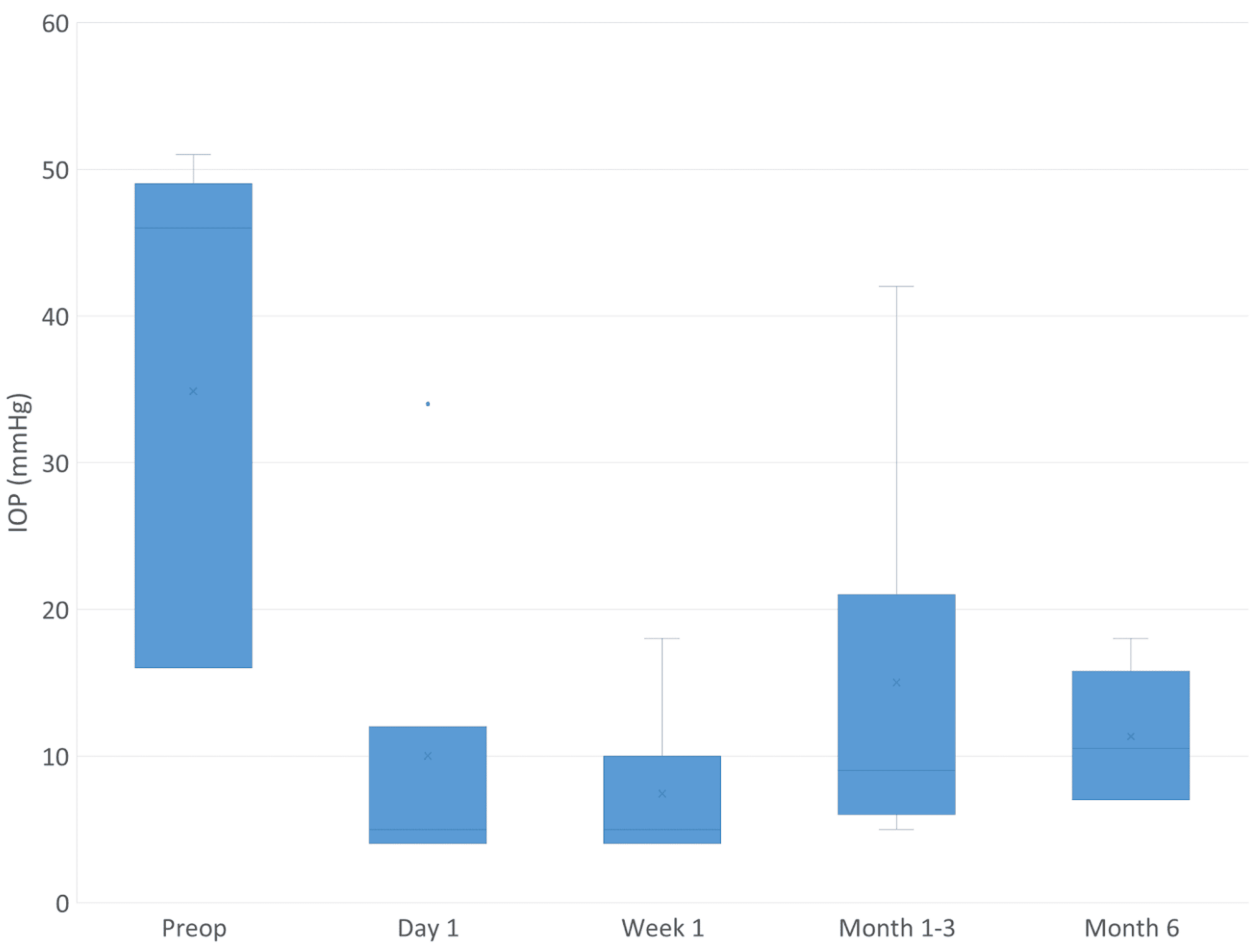
Intraocular Pressure (IOP) Reduction Over Time Line graph showing mean intraocular pressure (IOP) at baseline, day one, week one, month one, and month six following sutureless PreserFlo surgery with fibrin glue conjunctival closure (n=7). Error bars represent the standard deviation.

The mean IOP on day one post-operatively was 10.0±10.9 mmHg (Figure 2). Two isolated IOP elevations secondary to anterior chamber macrohyphema were observed in the NVG subgroup: one patient recorded 34 mmHg on day one, which resolved spontaneously to 18 mmHg by week 1 with conservative management; the second patient developed 42 mmHg at three months and required anterior chamber washout, after which IOP normalized to 7 mmHg by six months. Overall, three patients (two NVG, one POAG) developed macrohyphema requiring additional early follow-up (mean non-routine visits in the first month 3.7, range 3-4); two cleared spontaneously and one (NVG) underwent surgical washout within the first week.

At six months, all patients achieved a successful IOP reduction of >20% (mean, 11.3±3.8 mmHg). One patient resumed a single-glaucoma medication treatment. Visual acuities were stable in five patients and declined from hand movements and light perception to no light perception in two NVG patients diagnosed with Snuff syndrome in end-stage glaucoma. All patients were free of ocular pain at six months.

The conjunctival peritomy remained sealed in all cases. Three patients showed an uncomplicated recovery. One patient had transient hypotony, three had macrohyphema, and one (non-scleral band fixation group) had distal tube exposure managed by additional fibrin gluing. Fibrin glue-related adverse events were not observed. At six months, none of the scleral band fixation cases developed bleb-related complications or extrusion.

## Discussion

This case series reports the first use of fibrin glue for conjunctival closure in PreserFlo MicroShunt implantation combined with a novel scleral band fixation technique, demonstrating efficacy and safety in urgent glaucoma cases. This approach successfully addressed all the primary outcomes and offered a time-saving procedure.

Primary outcomes

All patients achieved >20% IOP reduction at six months (mean, 11.3±3.8 mmHg from 34.9±16.5 mmHg), meeting the criteria for success and similar to other PreserFlo MicroShunt studies reporting 10-15 mmHg postoperative IOP [1,4]. Ocular hypotensive medications decreased significantly from 3.3 (range 3-5) to 0.1 (range 0-1), reducing the treatment burden and consistent with the benefits of minimally invasive glaucoma surgery [5,6]. Non-standard postoperative reviews averaged 3.7 (range, 3-4), primarily due to macrohyphema in three patients. This review frequency is comparable to standard PreserFlo protocols, suggesting that the sutureless technique does not significantly increase early monitoring requirements [1,4].

Secondary outcomes

Complications included transient hypotony (n=1), macrohyphema (n=3), tube exposure (n=1), and Snuff syndrome (n=2, with pre-existing poor vision), similar to the known risks associated with PreserFlo surgery and unrelated to fibrin glue [1,4,7]. One patient required management of tube exposure with the use of additional fibrin glue. The absence of other glue-related complications supports the safety profile of fibrin glue as reported in a previous surgical series [2,3,8]. None of the scleral band fixation cases demonstrated extrusions or bleb-related issues [8].

VeraSeal fibrin glue, which combines fibrinogen and thrombin, ensures rapid tissue adhesion and reduces the operative time by eliminating suture placement [8]. Mean total operative time (skin-to-skin) for the seven sutureless cases using fibrin glue was 30.5±4.2 minutes compared with 55.7±5.1 minutes in the surgeon’s previous 14 consecutive PreserFlo cases requiring conventional conjunctival suturing. This efficiency is beneficial in emergency cases where rapid IOP control is desirable. The scleral band fixation, used in three cases, provided sutureless stability with well-formed, diffuse blebs, comparable to suture-based anchoring [9]. This approach minimizes conjunctival trauma and inflammation, enhances patient comfort, and reduces suture-related complications such as blebitis or endophthalmitis [10-12].

The efficacy of this technique in advanced glaucoma cases requiring emergency surgery was demonstrated by the successful reduction in IOP and resolution of ocular pain in all patients at six months, with five patients maintaining stable vision. The single-surgeon sutureless method streamlines workflow, reducing operative times and anesthetic exposure, particularly in elderly patients with comorbidities [13]. The absence of bleb leaks postoperatively underscores the reliability of fibrin glue in glaucoma surgery.

Several limitations should be acknowledged. Firstly, this preliminary series was limited by its small size (n=7), retrospective design, and lack of a control group with conventional sutured closure, limiting formal statistical analysis or direct comparisons with standard suture-based techniques. Secondly, follow-up was limited to six months, leaving longer-term outcomes unexplored. Furthermore, the single-centre, single-surgeon setting nature restricts generalizability. These limitations underscore the need for future prospective clinical trials comparing the use of fibrin glue versus sutured techniques.

## Conclusions

Fibrin glue is a safe and efficient method for conjunctival closure in PreserFlo MicroShunt surgery, eliminating sutures without compromising IOP outcomes, providing minimal non-routine post-operative reviews, and decreasing the use of glaucoma medications. The role of scleral band fixation in sutureless PreserFlo surgery and its effects on bleb formation require further evaluation. Further studies are recommended to investigate sutureless PreserFlo surgery using fibrin glue in broader clinical settings.
